# The Patient Flow Effect of Pandemic Policies: A Hybrid Simulation Study in a Norwegian Emergency Department

**DOI:** 10.3390/healthcare11010001

**Published:** 2022-12-20

**Authors:** Gaute Terning, Eric Christian Brun, Idriss El-Thalji

**Affiliations:** 1 Department of Safety, Economics, and Planning, University of Stavanger, 4036 Stavanger, Norway; 2 Department of Mechanical and Structural Engineering and Materials Science, University of Stavanger, 4036 Stavanger, Norway

**Keywords:** healthcare, emergency department, patient flow, computerized simulation modeling, agent-based hybrid model, multi-agent hybrid model, pandemic decision support

## Abstract

The COVID-19 pandemic required several interventions within emergency departments, complicating the patient flow. This study explores the effect of intervention policies on patient flow in emergency departments under pandemic conditions. The patient flow interventions under evaluation here are the addition of extra treatment rooms and the addition of a waiting zone. A predeveloped hybrid simulation model was used to conduct five scenarios: (1) pre-pandemic patient flow, (2) patient flow with a 20% contamination rate, (3) adding extra treatment rooms to patient flow, (4) adding a waiting zone to the patient flow, (5) adding extra treatment rooms and a waiting zone to the patient flow. Experiments were examined based on multiple patient flow metrics incorporated into the model. Running the scenarios showed that introducing the extra treatment rooms improved all the patient flow parameters. Adding the waiting zone further improved only the contaminated patient flow parameters. Still, the benefit of achieving this must be weighed against the disadvantage for ordinary patients. Introducing the waiting zone in addition to the extra treatment room has one positive effect, decreasing time that the treatment rooms are blocked for contaminated patients entering the treatment room.

## 1. Introduction

The COVID-19 pandemic has undoubtedly put a vast strain on healthcare systems worldwide. There is no doubt that the pandemic situation has had a big influence on how we now live our lives. This change includes the hospital emergency departments (EDs), which had to adjust their patient flow policies to accommodate patients with the risk of carrying the virus known for its high contamination rate [1,2]. The wake of the covid pandemic prompted the ED to face the fact that patients may be carrying the virus, thus putting staff and other patients at imminent risk. This risk was something that ED managers had to mitigate to the best of their ability. While in an unprecedented demanding situation, ED managers had to adjust the patient flow (PF) operation to adapt to several government-imposed restrictions, such as social distancing, the reduction of personal contact, personal protective equipment, and extra hygiene precautions, with other institution-specific guidelines [3,4].

The COVID-19 pandemic, characterized by its high rate of mutations, high transmissibility, and danger upon individuals with a low or compromised immune system, such as patients in an emergency department, has revitalized the need for flexible evaluation of interventions before implementation at low-risk and cost. As the onset of the COVID-19 pandemic emerged, patient pathways in EDs increased in complexity, leading to a need to rethink and evaluate the clinical patient flow pathway in patient handling [5].

In a complex system such as the ED, it is difficult to estimate the immediate and system-wide consequences of such changes on the overall patient flow [6,7]. Therefore, it is a good idea to use a low-cost and low-risk methodology to test the consequences of interventions intended to minimize the COVID-19 impact, i.e., computerized simulation modeling. Simulation studies appear to serve great potential in evaluating and foreseeing the patient flow implication of interventions in healthcare systems [8,9,10,11].

The patient flow in emergency departments has been studied through several approaches, such as field experiments, surveys, and simulations. One such simulation approach is called “in situ” simulation [12], in which team members of a patient care unit are physically involved in the simulation activity. Although beneficial in many ways, this mode of simulation can be resource- and time-demanding as it occupies operational resources. As an alternative, computer-based simulation modeling [12,13] has become increasingly popular. The study reported in this paper belongs to the latter category. Introducing new layouts, operating policies, and technologies to enhance the performance of emergency departments reinforces the need for a simulation modeling approach. This approach can predict ED behavior under introduced changes, such as layouts, policies, and technologies, without the need to implement them on a real scale [14]. Salmon et al. reviewed the simulation models applied to study emergency department issues and defined the common patterns and emerging trends. This review concluded that the discrete event approach was dominant in studying process performance, resource capacity, and workforce planning issues within emergency departments [13]. This conclusion was also confirmed by Hamza et al., as they found that few simulation models have examined the operational patient throughput [15]. Moreover, Salmon et al. highlighted that hybrid modeling (where different modeling approaches are combined) is limited and associated with a more strategic outlook [13]. However, hybrid modeling has huge potential to be used for operational aspects, as was proven by Hamza et al., where discrete-event and agent-based modeling were utilized to simulate the operational patient throughput [15].

Since early 2020, the COVID-19 pandemic has introduced pressures on the capacity of the emergency departments, and new policies (testing, prioritization, treatment) were applied. However, the literature that falls into the search “ALL = (emergency-department* AND simulation AND patient-flow* AND (pandemic OR covid*)” in the global citation databases Web of Science provides seven published contributions in total, with only three relevant ones [16,17,18,19]. Tavakoli et al. simulated the effect of patient flow during pandemics. They found that the studied ED might collapse after 14 days [16]. Bovim et al. simulated the ED patient flow during the pandemic. They found that the strict testing policy increases the bed requirements while having a minor effect on the number of ambulances [18]. Louhab et al. built a hierarchical-colored Petri Net model to simulate the patient flow in a restructured emergency department during the COVID-19 pandemic [19].

In the case of the ED in the Stavanger University Hospital, other policies were introduced in the emergence of the pandemic, including prioritizing the COVID-19-contaminated patients, applying extra treatment rooms, and applying waiting zones. Prioritizing the COVID-19-contaminated patients to enter the treatment rooms created a complex patient flow within the studied ED, as the normal patient has to leave the treatment room and move to the waiting zone once a COVID-19-contaminated patient arrives. The prioritization policy has also introduced the need for extra treatment rooms and waiting zones. Therefore, there was an urgent need to understand and examine the effect of these three policies (prioritization, extra treatment rooms, and waiting zones) on the overall patient flow. A hybrid simulation model was developed and published by Terning et al., where patient flow was simulated under pandemic conditions, prioritized COVID-19-contaminated patients, and introduced extra treatment rooms and waiting zones [20]. In this present study, the authors aimed to explore the effect of introducing extra treatment rooms and waiting zones on patient flow in emergency departments under pandemic conditions. The purpose of this study was to pose the following two research questions:

- How does the introduction of COVID-19-contaminated patients affect the patient flow in an emergency department?
- What effect does adding a waiting zone and extra treatment rooms have on patient flow in an emergency department?

Two sets of simulation experiments were planned to answer these two research questions. First, two simulation scenarios were performed to compare patient flow with and without COVID-19-contaminated patients to capture the effect of prioritizing the COVID-19-contaminated patients. Second, three simulation scenarios were performed to compare patient flow under three intervention policies: (1) by only adding extra treatment rooms, (2) by only adding a waiting zone, (3) by adding both extra treatment rooms and a waiting zone. Thus, in total, five simulation scenarios were designed and performed. Time to treatment, the average length of stay, crowding, and treatment room seizing frequency were the main patient flow performance indicators we estimated and compared. To perform these five scenarios and estimate the main patient flow performance indicator, the simulation model built by Terning et al. was used, where the emergency department of Stavanger University Hospital, a public hospital located in the southwest of Norway, was modeled [20].

The manuscript is divided into four main sections. In this section, Introduction, the subject of interest was contextualized, explained, and pointed to the two-fold aim this study pursued. In the following section, Section 2, the case study (layout and operations) and data sources (descriptions, patient arrival rates), simulation methodology (to restructure ED layout), the simulation model, the five designed simulation scenarios, and associated indicators are well described. In the penultimate section, Section 3, the simulated results of the five designed scenarios are summarized, visualized, and discussed. Additionally, the research contribution that discusses and validates the results of this study with results from previous studies found in the literature and identified research gaps for further work are provided. The last section, Section 4, answers the two research questions with concluding remarks on the overall benefits, challenges, and methodology used in this study.

## 2. Materials and Methods

This section presents the underlying simulation modeling methodology of the research project from which this paper presents its results. In addition, the section explains the data used to conduct the research. Lastly, a detailed overview of the overall scenario design is given that structures the research and results.

### 2.1. Case and Data Description

Our study was undertaken using the emergency department (ED) of the Stavanger University Hospital in Norway as our case. The hospital’s ED serves approximately 35,000 patients annually, i.e., roughly a hundred daily. Under normal conditions, i.e., prior to the COVID-19 pandemic, the case ED was equipped with 13 treatment rooms, 11 triage beds, and 7 assigned medical doctors [21,22,23,24].

**Figure 1:**
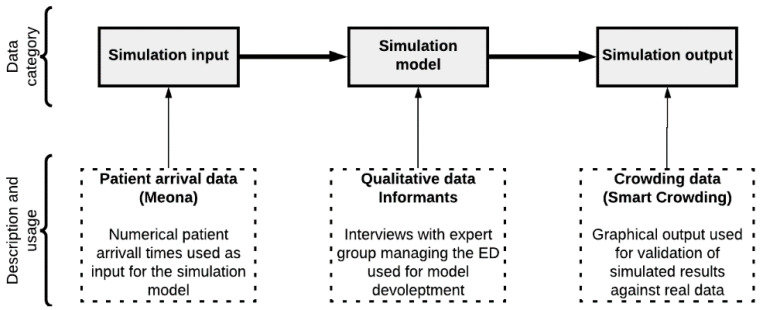
The data categories used in constructing, running, and validating the simulation model.

We negotiated access to the case with key personnel at the hospital’s analysis and research departments. Figure 1 shows an overview of the three categories of case data we were provided and used in our study.

**Figure 2:**
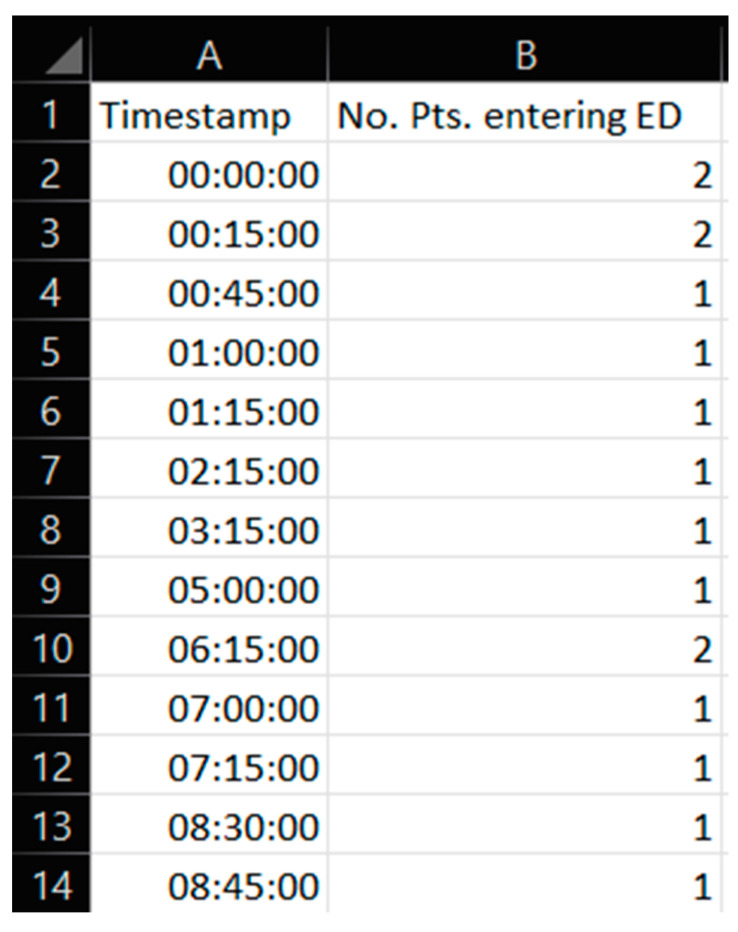
Screen capture from Meona showing patient arrival time data.

The first category was data used as simulation input. This category consisted of anonymized registrations of actual arrival times for patients at the ED. These data were recorded independently of and prior to this study. The dataset we were given access to was collected from a local database system called Meona. Each record of data in Meona showed only the number of patients that had arrived at specific time points during two specific days. No personal data were involved. All issues related to research ethical approval for our study were discussed and clarified with key staff responsible for overlooking research ethics at the case hospital. Given that our input data did not involve human subjects or personal data, our cooperating case hospital staff assured us that seeking patient consent was not relevant nor required for our study. An example of a series of Meona records is shown in Figure 2.

Meona thus provided information on the total number and distribution of patients’ arrivals on a particular day. The record of Meona data we used in our study spanned over two full, separate working days at the hospital (i.e., not in sequence) in the pre-pandemic situation, i.e., prior to the COVID-19 pandemic onset.

**Figure 3:**
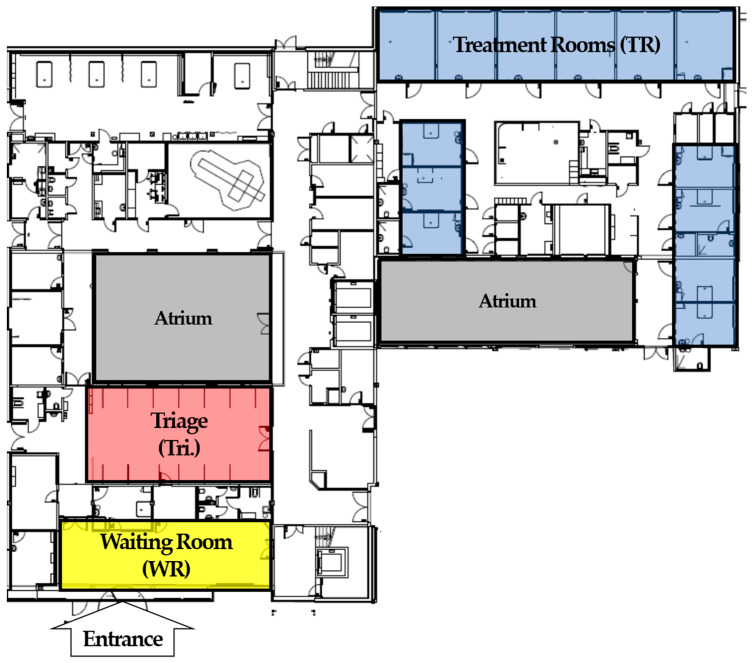
The blueprint of the studied ED prior to the onset of the COVID-19 pandemic. Entrance marked in green, waiting room in yellow, and treatment rooms in blue.

The second category of data was information about the patient flow process in the ED, which was the information we used to develop the simulation model. Such information included descriptions of the steps in the patient flow process and the criteria for deciding alternative routes through the system. This information was gathered through interviews and discussions with key personnel who worked closely with the ED on a daily basis. We were also given a blueprint of the layout of the ED, shown in Figure 3.

To compare the results of our simulations with the real-world data and thus verify and validate our simulation model, we used data retrieved from a system at the hospital called SmartCrowding. The SmartCrowding data consisted of graphs showing how the actual patient flow in the hospital’s ED developed during a given day. These data were represented through several performance indicators, for example, what time of day patient crowing reached a certain percentage of room capacity, peak crowding level, etc.

SmartCrowding data were not used in the study reported in this paper but were used in our preceding work to develop and validate our model, as reported in a previous paper [20]. In the study reported in this paper, the validated model resulting from our earlier work was used to simulate the effects of interventions (see Section 2.2.1) introduced to improve patient flow during the COVID-19 pandemic.

### 2.2. Simulation Modeling Methodology

**Figure 4:**
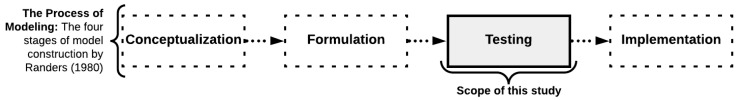
The Randers’ modeling methodology.

The computerized simulation model in this present research was built upon the Randers simulation modeling process. In a previously published paper [20], we reported the modeling process we went through to build our model and described how the model’s output parameters are calculated. Randers divides the process of simulation modeling methodology into a process of “conceptualization”, “formulation”, “testing”, and “implementation” [25]. In contrast to the previous study, which this study built upon, this present study’s scope falls entirely within the third step of the modeling process called “testing”, depicted in Figure 4 [20,26].

#### 2.2.1. The Introduced Interventions in This Case

To cope with the pandemic, the leaders of the case ED implemented several interventions; some interventions were resources, and some were policies on patient flow. These interventions were all implemented simultaneously as a package of interventions to cope with the pandemic.

- Pre-triage
  The pre-triage is an intervention in the form of a physical building in front of the ED entrance. In broad lines, patients must undergo screening by answering questions concerning the risk of contamination and taking a rapid antigen test.
- Fast-tracking
  They are expediting contaminated patients to treatment rooms. Patients that are found to have the risk of being contaminated are expedited directly to their designated treatment room. Other patients have to go by the normal procedure, wait in the waiting room and perhaps go through the triage; patients with the risk of being contaminated will be sent directly to their treatment room.
- Extra treatment rooms:
  To balance out the demand implications that the interventions put on the ED, they chose to increase the capacity of the ED by four rooms, each with its own bed. The balancing of capacity was performed by increasing the number of treatment rooms, which was 13 before the pandemic. The emergency department manager opens up four extra treatment rooms when needed.
- Waiting zone
  Another balancing intervention is the introduction of a waiting zone. The waiting zone is an area dedicated to non-contaminated patients who have received treatment and whose condition has stabilized. The need for treatment is reduced; thus, they can be placed in a new location. The policies of using the waiting zone for a patient are first that there are no more treatment rooms left over for a newly admitted patient and that the patient has received at least 2 h of care in their initial treatment room.

**Figure 5:**
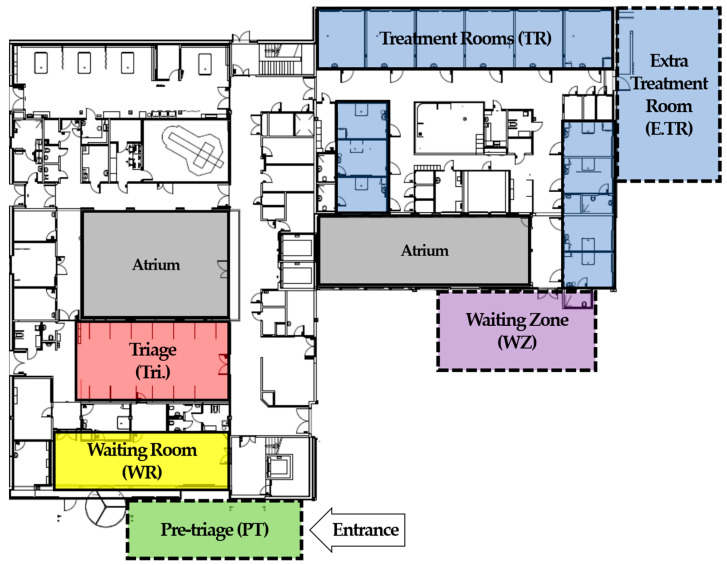
Case ED layout with the new pandemic introduced resources inside dotted lines. Waiting room (WR) in yellow, treatment rooms (TR) and extra treatment rooms (E.TR) in blue, pre-triage (PT) in green and waiting zone (WZ) in purple.

The layout of the particular case ED is an integral part of the modeling process. Figure 3 shows the normal layout under which the patient flow occurs. Under normal, pre-pandemic conditions, a patient enters the ED by going through the entrance area. After registering, the patient either waits in the waiting room or goes to the triage, depending on the patient’s status and the ED’s state. When the treatment room and priorities are over with, a patient goes to a treatment room and stays there until treatment is done and the patient is ready to be discharged from the department. After the aforementioned interventions, the ED is functioning as the configuration depicted in Figure 5. The figure shows the added resources in dotted lines, including treatment rooms (Extra Treatment Room, turquoise) and a zone where treated patients can stay (Waiting Zone) located underneath the treatment rooms.

### 2.3. Simulation Model

The computerized simulation model used to conduct the research is a so-called hybrid simulation model, which means that the model combines two or more simulation paradigms in simulation model construction [27]. It was acknowledged in previous research [28] that combining simulation model paradigms can produce a more accurate and reliable model. In this research, it was found during the simulation model-building process that it is necessary to combine paradigms in order to fully encompass the increased complexity that COVID-19 imposed on the clinical patient flow in the case emergency department. The model utilized modeling aspects found in the discrete event simulation (DES) paradigm and the agent-based modeling (ABM) paradigm. The two paradigms, DES and ABM, were intricately interweaved in the model by several interfaces; however they were roughly divided in two majors portions. The resource handling (i.e., treatment rooms, waiting zone spots, triage beds), patient inflow, and patient agent pathway movement across the layout is governed by the DES portion of the model, while the patient prioritization logic is governed by the ABM portion of the model. This delineation is in more detail elaborated in the previously disclosed research [21].

**Figure 6:**
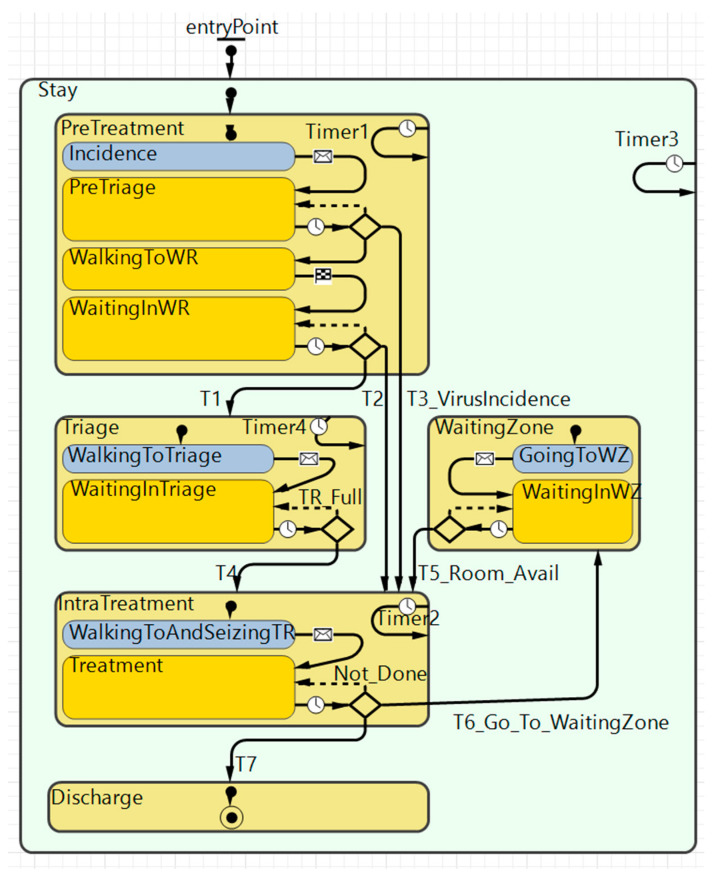
Statechart; the entity that governs the patient flow for every patient agent in the model.

The computerized simulation model utilized in the research was constructed using AnyLogic 8 Personal Learning Edition 8.8.1–Build 8.8.1.202210270952 x64 on a Personal Learning Edition License developed and distributed by the AnyLogic Company. This software was chosen because it incorporates the ability for hybrid modeling. The model in this work was validated and verified through previously disclosed work [20]. The simulation model is a hybrid model that combines aspects both from discrete-event- and agent-based modeling paradigms [29]. To implement the introduced prioritization of contamination-suspicious patients, we used a statechart, a feature of the agent-based modeling paradigm, as it specifies the specific logic of each patient agent during the model runtime. The statechart used in our simulation model is illustrated in Figure 6, and its underlying algorithm is described in a previous paper by the authors [21].

### 2.4. Scenario Design

At the onset of the COVID-19 pandemic in the general population, and thereby the onset of introducing potential virus-contagious patients to the ED, it was decided to implement a package of initiatives, hereafter called interventions, in order to respond to and minimize the risk represented by those patients that carry the contagion with them into the ED. The scenarios constructed in this simulation model were built to most accurately reflect the three main interventions that were implemented in the ED at our case hospital, namely the introduction of a patient prioritization system of suspected contaminated patients, extra treatment rooms, and the introduction of a waiting zone. These three interventions form, in total, five different scenarios, which are listed in Table 1 below.

One of the strengths of having a simulation model is the ability to run several different scenarios, making an informed analysis of what might happen if certain things were different. We can compare and contrast how performance will be under certain circumstances, such as policies, infection rate, etc. By doing this, we can answer particular “what-if” questions that can be insightful for policy recommendations and attempt to foresee their overall impact on a greater system. In this research, we ran scenarios designed to understand the various interventions’ contribution to the overall patient flow.

The way of answering these “what-if” questions here is to observe the differential outcome between the scenarios. Firstly, the base case scenario is constructed to reflect the situation that existed during the time of the data. The base case then serves as a reference for subsequent scenarios.

Sc. No.—Scenario number, PCR—patient contamination rate, P.T.—Pre-triage, E.Tr.—Extra treatment rooms, WZ—Waiting zone.

#### 2.4.1. Patient Flow Indicators

Several factors come into play in evaluating the patient flow of any ED. A betterment of one particular metric may come at the cost of other metrics. This simulation model study implemented several interventions to ensure most aspects concerning patient flow. The used patient flow indicators serve as an operationalization of PF and a basis for comparing the scenarios.

Table 2 presents an overview of the interventions introduced in the scenarios and an overview of the patient flow indicators that the simulation model calculated. A description and calculation formulae of each patient flow indicator are given in [20].

#### 2.4.2. The Data

The present study made use of a model previously developed specifically for the current case hospital emergency department. The simulation model’s development is disclosed in a previous paper [20], where the model is also validated through data and knowledgeable system informants. Thus, the model is primary data representing a major portion of the overall data utilized in this study. The patient flow data used for this study consist of the arrival times of patients to the ED from two representative days for the pre-pandemic situation. Although the previous paper used pre-pandemic and pandemic data for validation purposes, this present paper used the pre-pandemic to investigate the hypothesized outcomes before implementing patient flow interventions. Using this data allows for hypothesizing the pandemic interventions’ implication on the regular patient flow from the pre-pandemic time.

## 3. Results and Discussions

This chapter is structured in two main parts: First, in Section 3.1, we present the results from the simulations and discuss them scenario by scenario. This section of the paper shows the numerical results from the simulation model. While this section mostly compares scenarios one by one, a complete list of all the graphical outputs is found in the table placed in the Appendix A, Table A1.

Second, in Section 3.2, we discuss our research contribution relative to other comparable published research and discuss avenues for further research.

### 3.1. Presentation and Discussion of Simulation Results for Sc.1–Sc.5

From the output graphs in Table 3, we see that day two is considerably busier than day one, with a peak of about 45 patients, and day one, with a peak of about 35 patients. This observation fell along with expectations of what we could read from the data prior to scenario runs, as Day 1 had recorded 104. In contrast, Day 2 totaled 121 patients admitted to the ED, constituting a 16.3% increase in patient admission from day one until day 2.

When we look at the output values collected in Table 4, we can see the crowding from the two different days. The two most noticeable differences are the amplitude, i.e., the height of the curves, and the tail following the peak. 

Next, the following subsections summarize and compare all the individual scenarios.

#### 3.1.1. Scenario 1: The Base Case Scenario

The starting point for the scenario testing was running the base case scenario, which served as the reference point for all the other scenarios in this simulation study. As detailed in the scenario design, Table 1 on p. 8, this scenario was without any of the newly allocated resources. This scenario represented the situation before COVID-19 in the country; thus, there were no patients categorized with virus suspicion nor any extra treatment rooms or a waiting zone. Selected key patient flow indicators in this scenario are shown in Table 5.

In this simulation run, the average treatment time was 0.674 h and 1.096 h for Day 1 and Day 2, respectively. The ALOS was 2.299 h and 3.121 h. Table 6 shows the time development of patient prevalence in the ED during the days.

#### 3.1.2. Scenario 2: Pandemic: Introducing the Virus-Suspicious Patients, but No Interventions

Sc.2 introduces virus-contaminated suspicious patients, denoted “Con”, and the necessary policies needed to minimize cross-contamination risks within the ED. The main difference from the previous scenario is that this scenario introduces the prioritization of patients and, thus, patient flow patterns depending on the patient’s status. All the ordinary patients denoted “Ord” follow the patient flow pathway in the previous scenario. However, the patients found to be virus contamination-suspicious follow an alternative patient flow suited to the pandemic restrictions. Selected key patient flow indicators from running Scenario 2 are displayed in Table 7.

Compared to the output of Scenario 1, we see that the pandemic scenario at 20% PCR with no added resources shows, for the entire patient group, a higher total time to treatment (TtT = 0.896/1.124), a higher average length of stay (ALOS = 3.004/3.261), increased crowding (=47.207/60.883), increased peak crowding (=29/43). Additionally, there were more instances where the treatment room was blocked (=13/18) than in the pre-pandemic (with TtT = 0.674/1.096, ALOS = 2.699/3.121, crowding = 42.379/58.518). These patient flow indicators show that the virus-suspicious patients offset these measures significantly. Table 8 shows the time development of patient prevalence in the ED during the days.

As we can see, a further complication is the number of times the virus-suspicious patients have to wait for a treatment room to be available before they are isolated (13 on Day 1, 18 on Day 2). This blocking constitutes a big challenge and danger for cross-contamination within the ED, as fast-tracking the contamination-suspicious patients is crucial in reducing the risk.

Next, the three scenarios test the interventions that limit these adverse effects on the PF caused by suspicious virus patients: first, by using only extra treatment rooms; then, by using only a waiting zone; and finally, by using both the added extra treatment rooms and the waiting zone.

#### 3.1.3. Scenario 3: Pandemic and Introducing Extra Treatment Rooms

Selected key patient flow indicators in this scenario are displayed in Table 9.

Running this scenario highlighted the interesting observation that, despite the addition of four treatment rooms, which could increase patient prevalence in the ED of four, the peak crowding was reported from 29 to 24 on day one and 43 to 35 on day two. On the contrary, one could expect that the addition of treatment rooms would shift the peak in patient prevalence upwards by the same number of treatment rooms, as these four extra treatment rooms would allow the ED to hold more patients. However, this was not the case, as those treatment rooms increased the overall throughput, meaning the capacity to treat more patients and thereby reduce the peak in patient prevalence, as shown in the graphs. Thus, from an operational management perspective, even though four more patients theoretically could reside in the ED, the throughput effect associated with the E.TR. is greater than the possible accumulative effect.

By looking at crowding in Table 4, we see that adding the four treatment rooms reduced the number of patients residing in the ED. On day 1, crowding never surpassed 25% 20 pts; on day 2, the 30-pts threshold was only surpassed 7.1 % of the time. Table 10 shows the time development of patient prevalence in the ED during the days both for Sc.2 and Sc.3.

When comparing the scenarios for the same day, we see approximately that “Tr. in use” plateaus at coinciding times. By examining the output data in the graph, we observe that, for Day 1, “Tr. in use” plateaus at 13 patients at 11:33 in the morning, while for Day 2, it plateaus at 13 patients at 10:20. Logically, these times coincide with the “Time start use [when]” times for the triage for Scenario 1, as shown in Table 4.

#### 3.1.4. Scenario 4: Pandemic and Introducing the Waiting Zone as the Only Intervention

Sc.4 introduces the waiting zone where non-contagious patients are channeled in cases where they need to exit their treatment room to isolate contagious patients temporarily. Selected key patient flow indicators in this scenario are displayed in Table 11.

When comparing this scenario with Sc.2, we naturally see a marginal worsening across most patient flow indicators—except for time to treatment and ALOS for contaminated patients. This worsening is logical because non-contagious patients intermittently having to reside in a waiting zone increases their overall stay at the ED. Firstly, this is verified by looking at the ALOS, where the length of stay increased for the ordinary patient. In contrast, the length of stay decreased for contagious-suspicion patients. This decrease held both for day one and day two. Secondly, this is verified by looking at the increased peak crowding compared to Scenario 2.

On the other hand, times to treatment and ALOS improved, with an increase of 1 and 3 for contaminated patients 1 and 2, respectively. However, for the intention of this waiting zone in Sc.4 compared to Sc.2, one should also bear in mind that the intention of the waiting zone is not primarily to improve the patient flow, but it is to reduce the probability of cross-contamination of the disease. We can note that ALOS for the contamination-suspicion patients was reduced in Scenario 4 (with the WZ) compared to Scenario 2 (without the WZ). Hence, this reduction of time the contaminated patients reside in the ED may indicate that the risk of cross-contamination is also reduced. However, the biggest impact of the introduction of the WZ shows in the “Times TR blocked for cont.”, which reflected the real impact. By introducing the waiting zone on Day 1, the treatment rooms were only inaccessible for contamination-suspicious patients twice. In comparison, for Day 2, it was eight times, a relative reduction of 68% from Sc.2 for both days combined.

When comparing Sc.4 to Sc.3, we see that this scenario did not have the same beneficial impact on patient flow. Contrary to Sc.3., we here see that peak crowding is higher. Unlike Sc.3, the waiting zone does not increase the throughput of the ED. However, it merely increases how many patients can reside in the department. Table 12 shows the time development of patient prevalence in the ED during the days both for Sc.2 and Sc.4.

#### 3.1.5. Scenario 5: Pandemic and Using Extra Treatment Rooms and the Waiting Zone

This scenario includes the pandemic policies, the extra treatment rooms, and the waiting zone. Selected key patient flow indicators in this scenario are:

Looking at the intermediate scenarios, Sc.3 and Sc.4, Table 13 for Sc.5 and 3 are very similar, indicating that (20% contamination rate) as long as one has the presence of E.Tr., the addition of a WZ in Sc.5 did not make much difference to the total. The exception, again, is the improvement for contaminated patients and slight deterioration for ordinary patients as described above: time to treatment, TTT, for contaminated patients is lower when a WZ is present (0.029/0.033 in Sc.5 with WZ versus 0.054/0.212 in Sc.3 without WZ). ALOS for contaminated patients is slightly lower when a WZ is present (2.561/2.563 in Sc.5 with WZ vs. 2.585/2.743 in Sc.3 without WZ). However, the treatment room (TR) is blocked less often when a WZ is present (1/5 in Sc.5 vs. 3/22 in Sc.3).

An interesting point to discuss that may lead to controversy is when we compared the outcome of Sc.3 with Sc.5. Looking at Table 4, we see that ordinary patients considerably suffered after the introduction of the waiting zone. The ordinary patients here experienced worse treatment time and ALOS and the number of seizing was also larger. The only enhancement that Sc.5 provides, compared to Sc.3, is better ALOS for contaminated patients. Table 14 shows the time development of patient prevalence in the ED during the days both for Sc.2 and Sc.5.

#### 3.1.6. Effect on Time to Treatment (TtT)

Until now, the focus of the discussion was to compare the scenarios to their respective base cases. The next step in the analysis is to compare and contrast the key patient flow indicators to highlight their differential performance under the various interventions.

- When no interventions are introduced, and patients are routed through the ED according to contamination status, TtT for ordinary patients is much higher for ordinary than for contaminated patients.
- Introducing an extra treatment room (ET) causes a drop in TtT for both ordinary (Ord) and contaminated (Con) patients
- However, introducing the waiting zone (WZ), in addition to the extra treatment room, causes a further drop in TtT for contaminated (Con) patients but an increase in TtT for ordinary (Ord) patients.

The same pattern can be observed for the interventions’ effects on average length of stay (ALOS). Introducing the extra treatment room was beneficial for both ordinary and contaminated patients, while adding the waiting zone benefitted the contaminated patients but at the expense of added ALOS for ordinary patients. This observation, however, is not entirely surprising. The introduction of the extra treatment room implies an added treatment resource. Although it is reserved for the contaminated patients, it lessens the strain on the other treatment rooms, thus benefitting both contaminated and ordinary patients. Introducing the waiting zone, however, implies giving priority to contaminated patients. Ordinary patients will thus periodically experience that, on the incoming of a contaminated patient, their treatment will be halted, they will be put out to wait in a waiting zone, and they can only resume their treatment once they return. They will thus be subject to delays in their treatment, while the contaminated patients will experience being on a “fast track” through the system.

A similar observation can be observed in Table 4, showing the effect on crowding and peak crowding. However, these numbers show the total patient group results without distinguishing between ordinary and contaminated patients. Compared to a scenario of no interventions, adding the extra treatment room reduced crowding, while adding the WZ increased both crowding and peak crowding, possibly due to the effect mentioned above for ordinary patients.

An interesting effect can be observed by comparing the times the treatment rooms were blocked. While introducing the extra treatment room led to a clear improvement in TtT and ALOS but a less evident improvement in treatment room blocking (from 13/18 to 3/22), adding the waiting zone improved further (from 3/22 to 1/5).

#### 3.1.7. Summary of the Simulation Outputs

Introducing the extra treatment room led to a clear improvement in all the studied patient flow parameters. Adding the waiting zone further improved only the patient flow indicators related to the contaminated patients and had a negative impact on the overall and ordinary patient flow performance. The benefit of the waiting zone must be weighed against the disadvantage of this intervention for this intervention’s ordinary patients. In fact, ordinary patients are still better off in this situation than in a scenario with no interventions. Additionally our simulation output results indicated that the improved patient flow for contaminated patients provided by the waiting zone also helps limit cross-contamination. Furthermore, finally, introducing the waiting zone in addition to the extra treatment room has a clear positive effect on the times the treatment rooms are blocked.

### 3.2. General Discussion

To put the simulated outputs in context with other relevant studies found in the literature and clearly define the contribution, a general discussion is here made. Additionally, further research is recommended to level up the current state-of-the-art and continuously enhance the simulation modeling practices for patient flow in the emergency department under pandemic conditions.

#### 3.2.1. Research Contribution

This study has two main contributions: (1) studying a new set of resource-related policies (extra treatment room and waiting zone) on patient flow; (2) utilizing the hybrid modeling approach in a new context, i.e., pandemic patient flow. For the first contribution, it is clear that this study joined forces with previous studies such as Tavakoli et al. [16], Bovim et al. [18], and Louhab et al. [19] to address and provide insights about applying new pandemic-related policies that were not studied before, i.e., prioritizing the COVID-19-contaminated patients and adding an extra treatment room and waiting zone. Tavakoli et al. used a single paradigm modeling approach (discrete event simulation) to study when the ED might collapse, and no other patient flow intervention policies were applied. Bovim et al. utilized a multi-agent modeling approach (ED and ambulances) to study the effect of a strict testing policy on the bed and ambulances [18]. Louhab et al. built a hierarchical colored Petri Net model to simulate the patient flow in a restructured emergency department during the COVID-19 pandemic [19]. The study reported in this paper managed to mimic the general patient flow behavior (e.g., as shown in Table 3) in line with patient flow behaviors that were shown by Tavakoli et al. [16], Bovim et al. [18], and Louhab et al. [19]. However, the purposes (patient flow with and without interventions), the considered agents (patients, treatment rooms, beds, ambulances), and the studied behavior (testing policy, prioritizing policy, room seizing policy) of the studies are quite different and do not enable further comparison.

**Figure 7:**
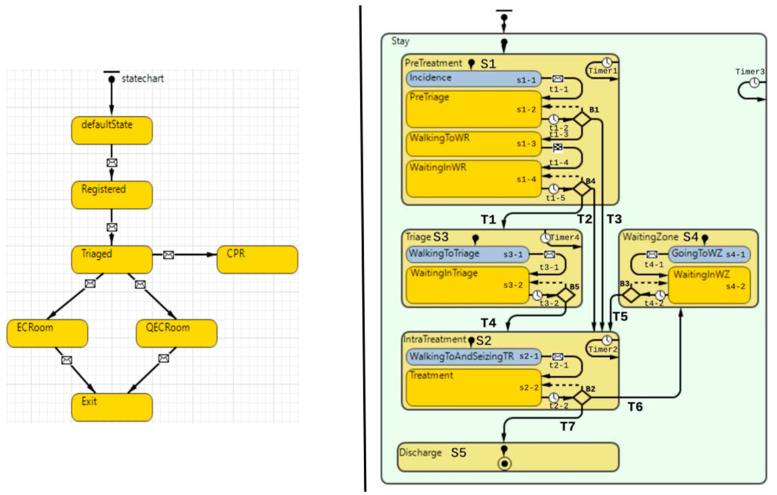
Juxtaposing the statechart used in a previously presented model in previous work [15] with the model used in this present study.

For the second contribution category, it is clear that this study contributed by utilizing the hybrid modeling approach and multi-agent approach and widened and extended knowledge and practices in modeling patient flow, which is still dominant with discrete-event modeling. In this sense, this study expanded the use of hybrid modeling under a non-pandemic situation, such as Hamza et al. [15], to a new context, i.e., a pandemic situation. By comparing the statechart model for non-pandemic patient flow, provided by Hamza et al. [15] and shown in Figure 7, with the statechart for pandemic patient flow, provided and used by this study, we clearly see how the presence of a pandemic causes a far more complex patient flow pattern. The research reported herein represents a contribution to meet the need for tools to handle and evaluate the added complexity in the clinical patient flow pathway caused by a pandemic such as COVID-19, as addressed by Capalbo et al. [5]. Additionally, this research showed an example of what type of output a hybrid model can produce. When used in decision-making, such an output can help decision-makers avoid potentially adverse effects from suggested interventions intended to improve patient flow.

Our research also represents a unique contribution to the previous models that covered the pandemic aspects of the healthcare sector so far have addressed issues such as virus spread and transmission [30] and vaccination [31], while studies that address patient flow under pandemic conditions have been sparse [32]. Additionally, Bhattacharjee and Ray call for future research on integrating and combining different patient flow modeling techniques [11], which we believe this paper provided.

#### 3.2.2. Further Work

In conducting the work presented in this manuscript, multiple avenues were identified for furthering knowledge on the multi-agent modeling of patient flow in emergency departments with pandemic intervention. Here, we list several recommendations to extend this present work as follows:

- Cleaning and disinfection time: When looking at the numerical output portrayed in Table 4, we can see the indicator of how many times a room was seized. Under pandemic conditions, a treatment room must be sanitized between each patient visit. This will not only need resources, but it will also take time. Different scenarios gave different seizing times, particularly Sc.4 and Sc.5, which included the waiting zone to be utilized. Implementing time for sanitization could reveal a further “cost” of including a waiting zone in the ED.
- Optimization of the number of extra treatment rooms: The results that are shown in Section 3.1. show favorable patient flow outcomes when including the extra treatment rooms. In order to make the model reflect the actual patient flow interventions that the case ED implemented, scenarios Sc.3 and Sc.5 included four treatment rooms. Since treatment rooms showed such an overall improvement in patient flow, it would be fruitful to investigate the optimal number of extra treatment rooms to add.
- Classification error: Similar to any classification system, pre-triage prescreening may be vulnerable to false positives and false negatives. Here, the risk associated with false negatives might have a devastating impact on a department filled with compromised patients. Such a case could imply contagious patients admitted to the waiting room.
- Studying the effect of the increased patient contamination rate on the patient flow: Given the results disclosed in this present article, an immediately succeeding logical step could be to investigate differential outcomes with an incremental increase of the patient contamination rate. Such a granular approach could evaluate and inform the intervention efficacy under different patient contamination rates and investigate and inform at which levels the non-contaminated patients have to suffer too great of a burden.
- Intra-hospital contamination: As shown in the literature on the current pandemic, we know that the pathogen is highly transmissible. There could be a risk of intradepartmental virus transmission if a patient is near another patient. Additionally, the screening may not be accurate, and patients might be contaminated. This is quite easy to implement in an agent-based model such as the one used in the present paper. Such an endeavor was pursued in the research of [33]. However, it is not clear if an actual patient flow moving across the physical layout was taken into consideration

## 4. Conclusions

This paper aimed to study various effects of COVID-19 pandemic interventions to be undertaken on the case organization’s patient flow in the ED in terms of operational impacts (time to treatment, the average length of stay, treatment room utilization, crowding, time spent in the waiting room, etc.). Thus, the operational impacts were analyzed through a case study, conceptualized, and computationally modeled to represent the operational behavior of patient flow in an emergency department that handles, together with the ordinary patient, suspected patients with COVID-19.

The main conclusion of this study is that the COVID-19 interventions complicate the patient flow in the emergency department in several means:

1. The suspected COVID-19 patient requires prioritization over the ordinary patient to the level that ordinary patients must leave the treatment room for suspected COVID-19 patients;
2. It increases the occupancy rate for treatment rooms and leads to more frequent seizing and releasing operations for treatment rooms due to the prioritization and random arrival rate of suspected COVID-19 patients;
3. It increases the need for additional services for treatment rooms and waiting zones;
4. It increases the lead time, treatment time, and waiting time for the patients not found to be contaminated.

Therefore, the effect of the pandemic situation on patient flow is captured in both the conceptual and simulation models.

The resulting simulation model can be utilized for several purposes: short and mid-term capacity planning and long-term layout redesign. We have become more aware of the pandemic behaviors and waves regarding short and mid-term capacity planning. The emergency department is usually interested in predicting the behaviors of their patient flow for each coming pandemic wave. Such predictions help them to plan their resources (medical doctors, nurses, cleaners, equipment, rooms) and apply the most effective procedure (regarding testing, treatment, overtime capacity, and shared/outsourced resources). It is not sufficient for the emergency department to predict how many virus-suspicious patients will arrive in the coming pandemic wave. They must also predict the daily arrival rate over a representative time interval.

Moreover, the emergency department shall also be able to predict other ordinary patients, as this also might vary over time and season, which might create a “tragedy of commons” situation, as almost the same limited resources serve all patient categories. Another important issue that should be noticed and can lead to the “Eroding goals” situation is the priority for virus-suspected patients over ordinary patients. This prioritization might reduce patient satisfaction, as the patient is asked to leave the treatment rooms and stay in a waiting room. In summary, the built simulation model effectively informs emergency department managers about when they should expect bottlenecks, high waiting time intervals, and which resources need to be leveled up and for how long. Additionally, they can devise temporary procedures to reduce the “tragedy of commons” and “eroding goals” situations.

## Figures and Tables

**Table 1:** Scenario descriptions and their respective configuration of resources and contamination.

Sc. No.	Explanation	Model and Resource Configuration			
		PCR [%]	P.T.	E.Tr.	WZ
Sc. 1	Base case, pre pandemic operation: This scenario sets the emergency department operation to the pre-pandemic situation, like it was during the time of data gathering. The extra treatment rooms and the waiting zone is not in use and none of the patients will be treated as being virus suspicious. Regular path of patient flow will be followed. This is before any implementation of any novel resources into the ED.	0			
Sc. 2	Scenario simulating situation during pandemic operation. However, no extra resources are introduced, only the new policies are introduced, channeling and expediting the contaminated patients according to the policies. Thus, the E.Tr. and WZ is not in use for this scenario. P.T. is necessarily introduced as it functions as the mechanism of sorting contaminated patients from the non-contaminated patients.	20	**✓**		
Sc. 3	Scenario simulating situation during pandemic operation with E.Tr. introduced. This scenario is made in order to isolate out the effect of the waiting zone that. Otherwise, all else is equal as Sc.2.	20	**✓**	**✓**	
Sc. 4	Scenario simulating pandemic operation with WZ introduced: The operation is set up as the case organization during the m, with the only change of excluding the extra treatment. Doing this, we can see how the operation of the ED would be without these four extra treatment rooms used.	20	**✓**		**✓**
Sc. 5	Scenario simulating pandemic operation. The operation is set up as the case organization during the pandemic situation. Doing this, we cans see the combined effect of the two policies.	20	**✓**	**✓**	**✓**

Sc. No.—Scenario number, PCR—patient contamination rate, P.T.—Pre-triage, E.Tr.—Extra treatment rooms, WZ—Waiting zone.

**Table 2:** Overview of terms used to track patient flow performance of the scenarios in this study.

Abbreviation	Description and Explanation	Unit
Resources:		
**PT**	Pre-Triage: A resource introduced for questionnaire and test of the admitted patients before entering the ED. From here patients are treated as either ordinary patients or contaminated patients.	-
**Tri.**	Triage: The place where patients normally, i.e. when not found to be contagious, will go after being admitted to the ED. Patients will wait here until a treatment room is available.	
**E.TR.**	Extra treatment rooms: These are treatment rooms put in addition to the existing treatment rooms in the ED. These are used to accommodate the other adverse effect that the pandemic pose to the ED.	-
**WZ**	Waiting Zone: A dedicated area for ordinary patients whenever they have to leave their treatment room to make room for a contaminated patient.	-
Model inputs:		
**OP**	Ordinary patients: Non-contaminated patients that in the pre-triage were not found to be carriers of pathogens.	#
**CP**	Contaminated patients: Patients that in the pre-triage were found to be contagious and carriers of pathogens and thus risk of	#
**PCR**	Patient contamination rate: A variable that is dependent on spread of infectious decease in the general population. In this study it is set to 0% and 20% according to the scenario design.	%
Patient flow indicators:		
**TTT**	Time to Treatment: The patient flow indicator calculates how long time on average it takes before the treatment of a patient gets started. Values are given in number of hours per patient.	Hours per patient [h/pt]
**ALOS**	Average length of stay—Calculates how long is the average time spent in the emergency department, from point of entry until	Hours per patient [h/pt]
**Crowding**	The crowding indicator tracks how many patient are prevalent in the department at any given time and whenever threshold is passed, the time duration the threshold has been surpassed is divided on the total running time. Crowding is based on the case organization own framework of managing patient flow; ‘Plan for high activity’. Which has defined three crowding levels relevant for the ED: 15, 25 and 30 patients each of which has their specifically tailored modus operandi.	%
**Peak crowding**	This indicator shows how many patients are prevalent on the busiest time of the day and it also keeps the time stamp of when this peak occurred	# & Time
**Time start use/Time in use**	This patient flow indicator shows when relevant resources (i.e. E.TR, Tri. and WZ) was first to be used by a patient during that day. A reading in the Tr.-column at ‘11:00’ means that the triage that day was not used until 11:00. Second value shows the percentage amount of that day this resource has been used at least by one patient.	Time
**Time full**	This patient flow indicator counts how long the different resources has been at their maximal capacity.	Time & %
**Time in WR**	This patient flow indicator calculates the average time patients use in the waiting room.	h
**Times TR blockedfor con.**	This measures counts the amount of times contaminated patients cannot get access to a treatment room.	#
**Times TR seized**	This measure counts how many times a treatment room is seized.	#

**Table 3:** Showing the patient crowding for the two representative days in a pre-pandemic setting.

	Day 1	Day 2 ^†^
Real data	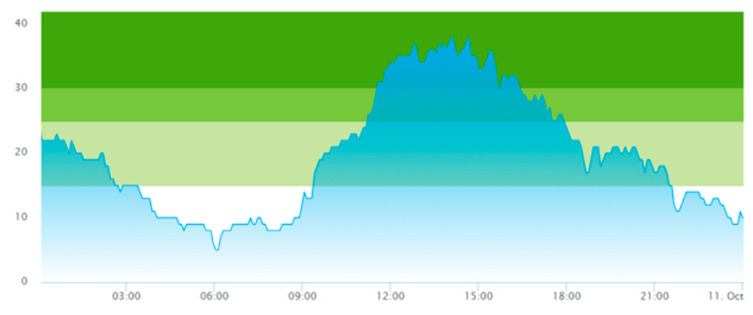	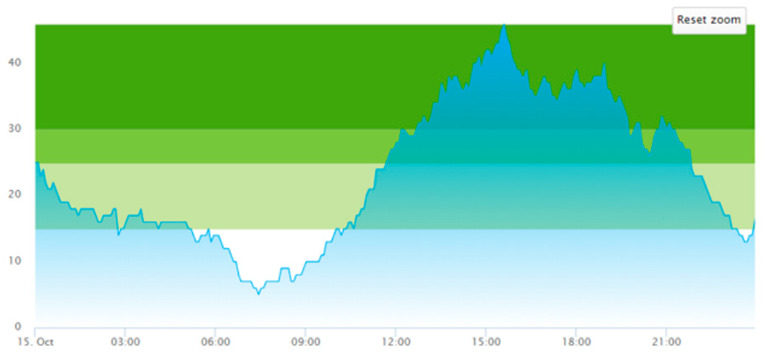

†—Graph ‘Day 2’ is not the immediately following day after ‘Day 1’ as the naming may suggest.

**Table 4:** Tabulated PF indicators for the simulation outputs of scenarios Sc.1–Sc.5.

Scenario No.		Time to Treatment [h/pt]			ALOS [h/pt]			Crowding [%] (Time Value Passed [When])			Peak Crowding ED [#] (Time of Peak [When])	Time Start Use [When] (Time in Use [%])			Time Full [When] (Time Full [%])			Time in WR (h/pt)	Times TR Blocked for Cont. (#)	Times TR Seized [#] (Times WZ Seized [#])
	Day	Tot.	Ord.	Con.	Tot.	Ord.	Con.	>15	>25	>30	ED	E. TR	Tri.	WZ	E. TR	Tri.	WZ			
**1**	1	0.674	0.674	null	2.699	2.699	null	42.379 (10:45)	20.182 (14:45)	0 (null)	26 (14:45)	null (0.0)	11:00 (52.876)	null (0.0)	null (0.0)	14:19 (4.697)	null (0)	0.034	0	124 (0)
	2	1.096	1.096	null	3.121	3.121	null	58.518 (11:45)	39.051 (14:45)	26.606 (15:00)	39 (17:15)	null (0.0)	11:33 (63.199)	null (0.0)	null (0)	12:48 (39.280)	null (0)	0.259	0	138 (0)
**2**	1	0.896	1.038	0.178	3.004	3.063	2.708	47.207 (10:45)	7.749 (14:15)	0 (null)	29 (14:45)	null (0)	11:00 (58.642)	null (0)	null (0)	14:18 (10.745)	null (0)	0.069	13	124 (0)
	2	1.124	1.372	0.255	3.261	3.397	2.785	60.883 (11:45)	41.164 (14:45)	32.268 (15:00)	43 (19:30)	null (0)	11:33 (64.901)	null (0)	null (0)	13:18 (40.597)	null (0)	0.261	18	132 (0)
**3**	1	0.203	0.231	0.054	2.311	2.258	2.585	29.420 (10:45)	0 (null)	0 (null)	24 (14:45)	11:00 (46.275)	12:03 (21.252)	null (0)	11:00 (28.675)	null (0)	null (0)	0.028	3	124 (0)
	2	0.714	0.878	0.212	2.865	2.905	2.743	47.828 (11:45)	23.168 (13:15)	7.106 (15:15)	35 (17:30)	11:33 (59.467)	12:18 (45.931)	null (0)	11:48 (48.140)	15:18 (10.606)	null (0)	0.081	22	146 (0)
**4**	1	0.979	1.181	0.030	3.094	3.208	2.560	53.000 (10:45)	7.777 (14:15)	0 (null)	30 (15:45)	null (0)	11:00 (60.379)	11:00 (16.165)	null (0)	14:18 (12.496)	null (0)	0.084	2	140 (16)
	2	1.212	1.604	0.039	3.367	3.633	2.568	61.392 (11:45)	43.797 (13:15)	35.687 (15:00)	46 (17:30)	null (0)	11:33 (64.683)	11:45 (26.540)	null (0)	13:03 (39.153)	null (0)	0.259	8	158 (25)
**5**	1	0.210	0.245	0.029	2.320	2.273	2.561	29.543 (10:45)	0 (null)	0 (null)	25 (14:45)	11:00 (46.277)	12:03 (21.085)	12:15 (4.964)	11:00 (31.066)	null (0)	null (0)	0.029	1	130 (6)
	2	0.845	1.134	0.033	3.006	3.164	2.563	49.997 (11:45)	26.996 (13:00)	11.996 (15:15)	39 (17:30)	11:33 (66.143)	11:33 (66.143)	12:15 (21.178)	11:48 (51.553)	15:03 (17.136)	null (0)	0.147	5	168 (22)

Tot.—Total patient population, Ord.—Ordinary patients without suspicion of being pathogen contagious, Con.—Patients with suspicion of being pathogen contagious, ED—Emergency department, E.Tr.—Extra treatment rooms, Tri.—Triage, WZ—Waiting zone, WR—Waiting room.

**Table 5:** Table displaying a subset selection of KPI from the Sc.1 output.

Key Patient Flow Indicator	Day 1	Day 2
Time to treatment (TtT)	0.674	1.096
Average length of stay (ALOS)	2.699	3.121
Crowding > 15%	42,379	58,518
Peak crowding	26	39
Times treatment room (TR) is blocked	0	0

**Table 6:** Simulation output crowding graphs for Sc.1.

	Day 1	Day 2 ^†^
(Sc. 1)	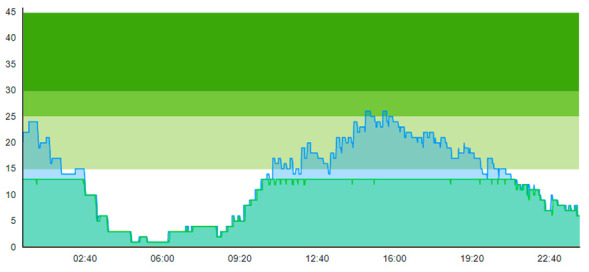	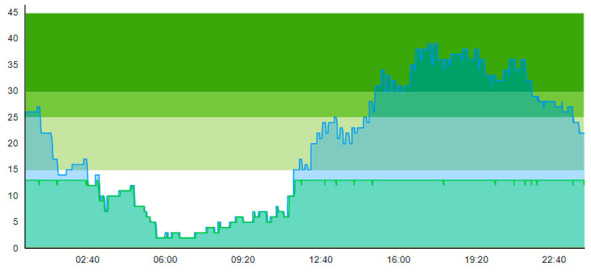
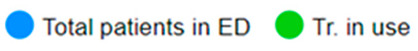		

†—‘Day 2’ are not the immediate successor following ‘Day 1’ as the naming may suggest.

**Table 7:** Table displaying a subset selection of KPI from Sc.2.

Key Patient Flow Indicator	Day 1			Day 2		
Patient Type:	All	Ord	Con	All	Ord	Con
Time to treatment (TtT)	0.896	1.038	0.178	1.124	1.372	0.255
Average length of stay (ALOS)	3.004	2.063	2.708	3.261	3.397	2785
Crowding > 15%	47.207			60.883		
Peak crowding	29			43		
Times treatment room (TR) is blocked	13			18		

**Table 8:** Simulation output crowding graphs for comparison between Sc.2 and Sc.1.

	Day 1	Day 2 ^†^
(Sc. 1)	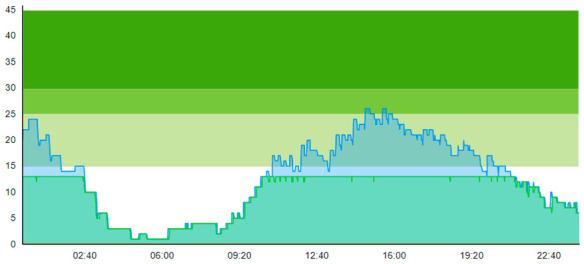	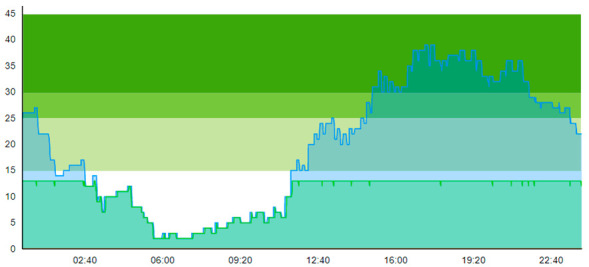
(Sc. 2)	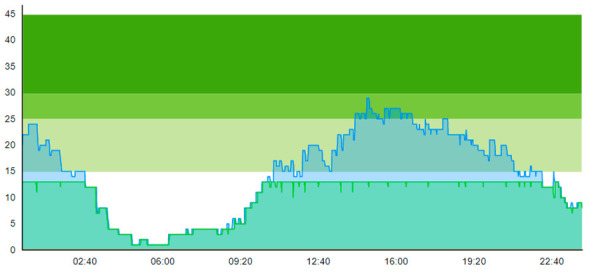	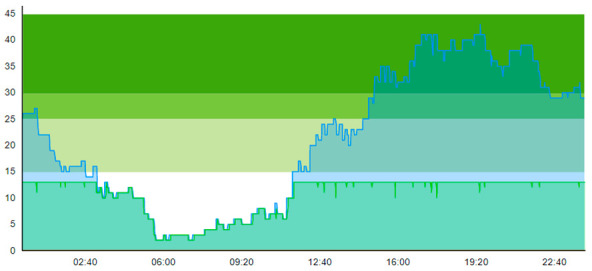
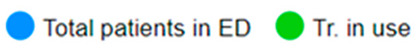		

†—‘Day 2’ are not the immediate successor following ‘Day 1’ as the naming may suggest.

**Table 9:** Table displaying a subset selection of KPI from Sc.3.

Key Patient Flow Indicator	Day 1			Day 2		
Patient Group:	All	Ord	Con	All	Ord	Con
Time to treatment (TtT)	0.203	0.231	0.054	0.714	0.878	0.212
Average length of stay (ALOS)	2.311	2.258	2.585	2.865	2.905	2.743
Crowding > 15%	29.420			47.828		
Peak crowding	24			35		
Times treatment room (TR) is blocked	3			22		
Times TR seized	124			146		

Adding the extra treatment rooms improves PF in nearly every parameter for both days, with the exceptions of ALOS for ordinary patients on Day 1 and times TR blocked on Day 2.

**Table 10:** Simulation output crowding graphs for comparing Sc.3 with Sc.2.

	Day 1	Day 2^†^
(Sc. 2)	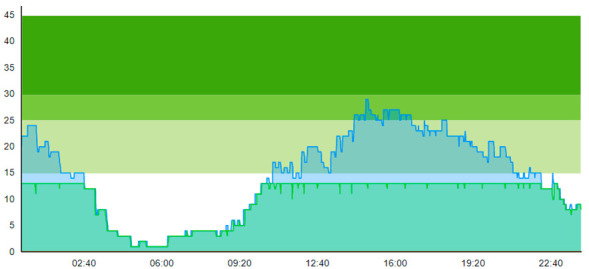	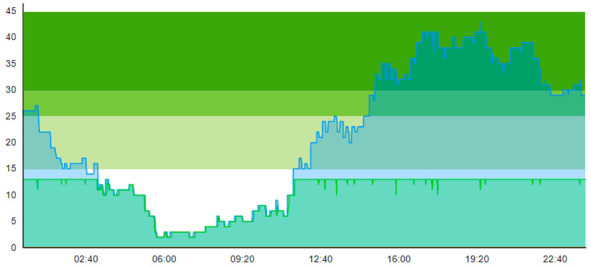
(Sc. 3)	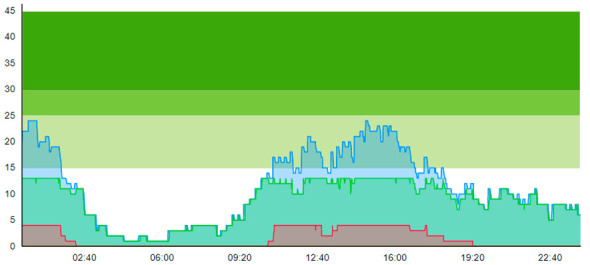	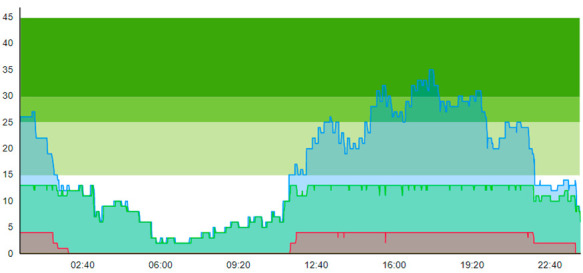
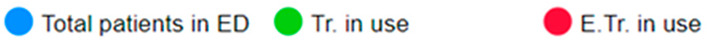		

†—‘Day 2’ are not the immediate successor following ‘Day 1’ as the naming may suggest.

**Table 11:** Table displaying a subset selection of KPI from Sc.4.

Key Patient Flow Indicator	Day 1			Day 2		
Patient Group:	All	Ord	Con	All	Ord	Con
Time to treatment (TtT)	0.979	1.181	0.030	1.212	1.604	0.039
Average length of stay (ALOS)	3.094	3.208	2.560	3.367	3.633	2.568
Crowding > 15%	53.000			61.392		
Peak crowding	30			46		
Times treatment room (TR) is blocked	2			8		
Times TR seized	140			158		

**Table 12:** Simulation output crowding graphs for comparing Sc.4 with Sc.2.

	Day 1	Day 2 ^†^
(Sc. 2)	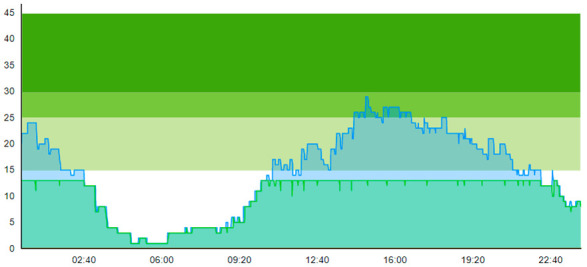	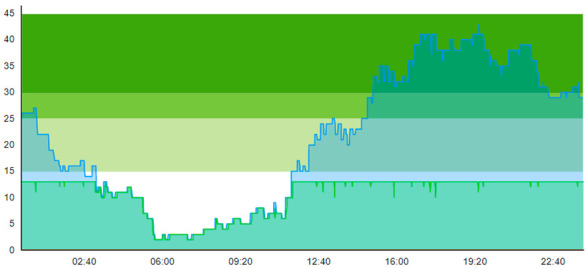
(Sc. 4)	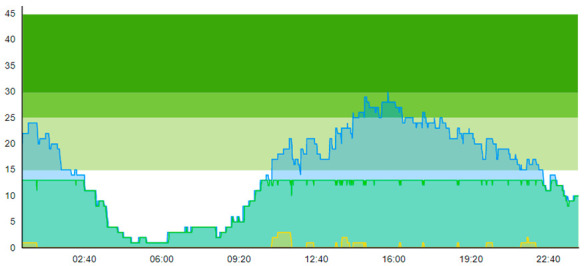	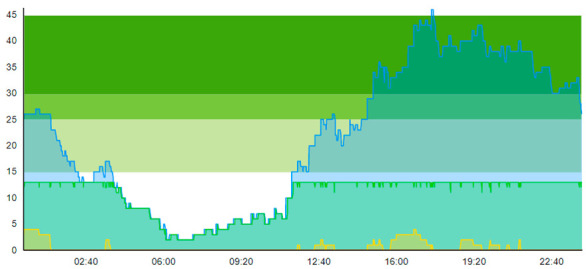
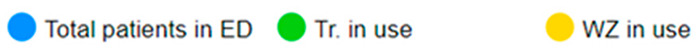		

†—‘Day 2’ are not the immediate successor following ‘Day 1’ as the naming may suggest.

**Table 13:** Table displaying a subset selection of KPI from Sc.5.

Key Patient Flow Indicator	Day 1			Day 2		
Patient Group:	All	Ord	Con	All	Ord	Con
Time to treatment (TtT)	0.210	0.245	0.029	0.845	1.134	0.033
Average length of stay (ALOS)	2.320	2.273	2.561	3.006	3.164	2.563
Crowding > 15%	29.543			49.997		
Peak crowding	25			39		
Times treatment room (TR) is blocked	1			5		
Times TR seized	130			168		

**Table 14:** Simulation output crowding graphs for comparing Sc.5 with Sc.2.

	Day 1	Day 2 ^†^
(Sc. 2)	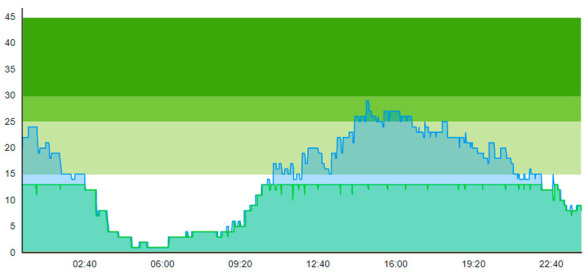	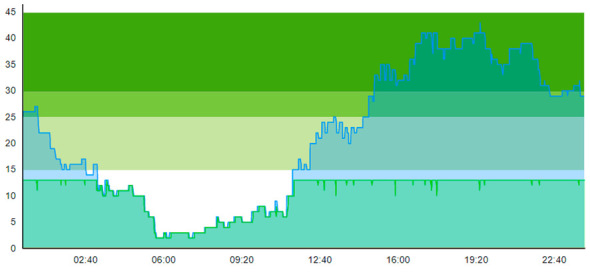
(Sc. 5)	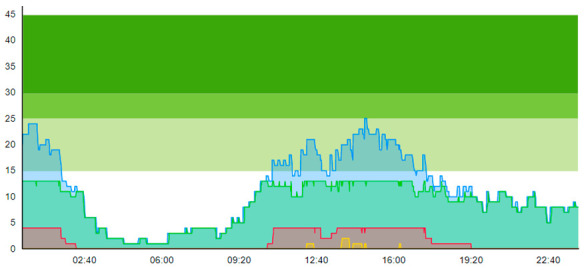	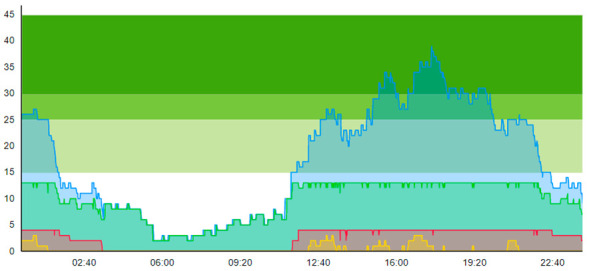
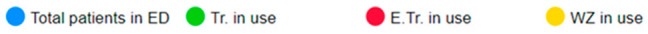		

†—‘Day 2’ are not the immediate successor following ‘Day 1’ as the naming may suggest.

## Data Availability

The numerical data used in this study are not publicly available and thus are not for distribution.

## Abbreviations

ABM—agent-based modeling, DES—discrete event simulation, ED—emergency department, PF—patient flow, SUS—Stavanger Universitetssykehus (The University Hospital of Stavanger), WZ—waiting zone, TR—treatment room, Tri.—triage, PT—pre-triage, E.TR—extra treatment room, WR—waiting room, PF—patient flow, PCR—patient contamination rate, TTT—time to treatment, ALOS—average length of stay.

## Appendix A

**Table A1:** Following is a tabulation of all the graphical output produced by running every scenario from 1 until 5.

	Day 1	Day 2 ^†^
(Sc. 1)		
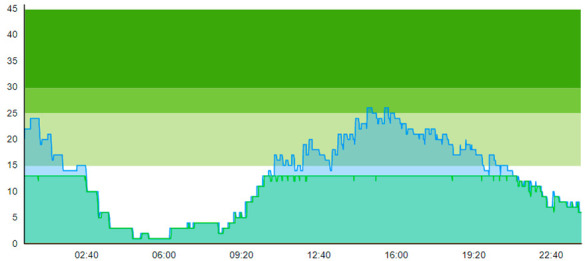
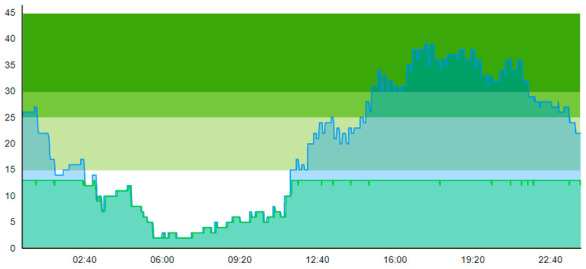
(Sc. 2)		
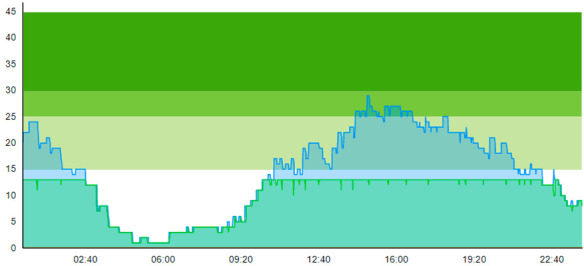
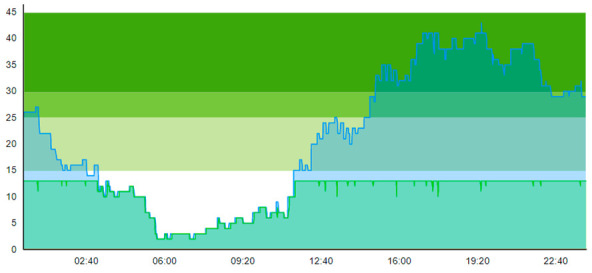
(Sc. 3)		
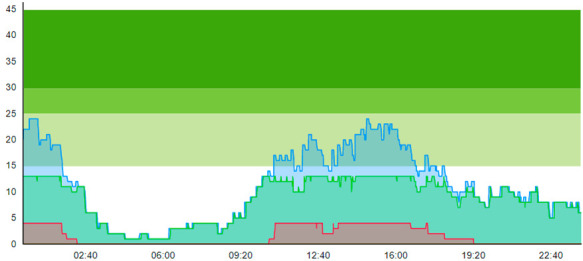
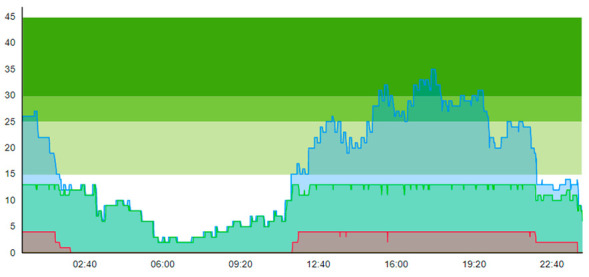
(Sc. 4)		
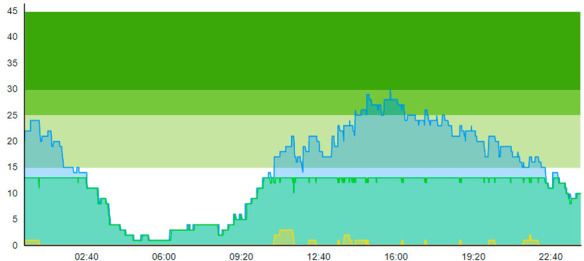
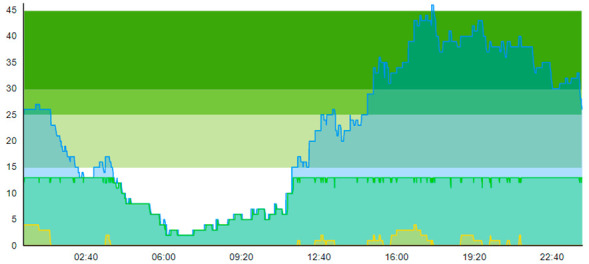
(Sc. 5)		
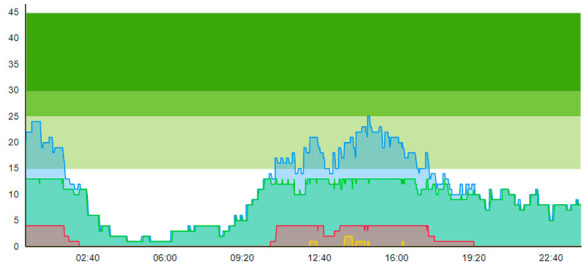
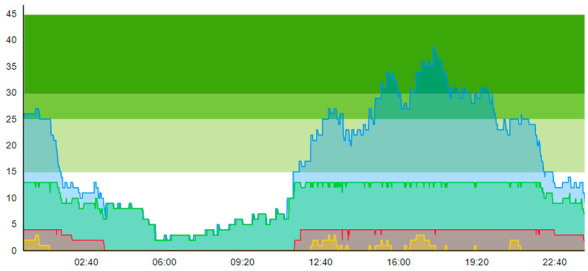

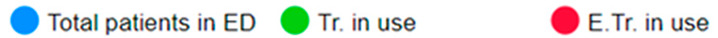

†—‘Day 2’ are not the immediate successor following ‘Day 1’ as the naming may suggest.

## References

[1] Ciotti M., Ciccozzi M., Terrinoni A., Jiang W.-C., Wang C.-B., Bernardini S. (2020). The COVID-19 Pandemic. Crit. Rev. Clin. Lab. Sci..

[2] Martellucci C.A., Flacco M.E., Cappadona R., Bravi F., Mantovani L., Manzoli L. (2020). SARS-CoV-2 Pandemic: An Overview. Adv. Biol. Regul..

[3] Helse Stavanger S. Koronavirus—Rutiner for Medarbeidere. https://helse-stavanger.no/om-oss/for-ansatte/koronavirus-rutiner-for-ansatte.

[4] Gro F. Håndteringen av COVID-19-Pandemien. 31. https://www.regjeringen.no/contentassets/dee8c25ba49f4e21add931746e04f7fb/regjeringens-strategi-og-beredskapsplan.pdf.

[5] Capalbo C., Aceti A., Simmaco M., Bonfini R., Rocco M., Ricci A., Napoli C., Rocco M., Alfonsi V., Teggi A. (2020). The Exponential Phase of the COVID-19 Pandemic in Central Italy: An Integrated Care Pathway. Int. J. Environ. Res. Public Health.

[6] Rutherford P.A., Provost L.P., Kotagal U.R., Luther K., Anderson A. (2017). Institute for Healthcare Improvement: Achieving Hospital-Wide Patient Flow.

[7] McHugh M., VanDyke K., McClelland M., Moss D. Improving Patient Flow and Reducing Emergency Department Crowding: A Guide for Hospitals. 48. https://www.ahrq.gov/research/findings/final-reports/ptflow/index.html.

[8] Mohiuddin S., Busby J., Savović J., Richards A., Northstone K., Hollingworth W., Donovan J.L., Vasilakis C. (2017). Patient Flow within UK Emergency Departments: A Systematic Review of the Use of Computer Simulation Modelling Methods. BMJ Open.

[9] Vanbrabant L., Braekers K., Ramaekers K., Van Nieuwenhuyse I. (2019). Simulation of Emergency Department Operations: A Comprehensive Review of KPIs and Operational Improvements. Comput. Ind. Eng..

[10] Bansal K., Kumar S. (2022). Mutational Cascade of SARS-CoV-2 Leading to Evolution and Emergence of Omicron Variant. Virus Res..

[11] Bhattacharjee P., Ray P.K. (2014). Patient Flow Modelling and Performance Analysis of Healthcare Delivery Processes in Hospitals: A Review and Reflections. Comput. Ind. Eng..

[12] Aljahany M., Alassaf W., Alibrahim A.A., Kentab O., Alotaibi A., Alresseeni A., Algarni A., Algaeed H.A., Aljaber M.I., Alruwaili B. (2021). Use of In Situ Simulation to Improve Emergency Department Readiness for the COVID-19 Pandemic. Prehosp. Disaster Med..

[13] Salmon A., Rachuba S., Briscoe S., Pitt M. (2018). A Structured Literature Review of Simulation Modelling Applied to Emergency Departments: Current Patterns and Emerging Trends. Oper. Res. Health Care.

[14] Castanheira-Pinto A., Gonçalves B.S., Lima R.M., Dinis-Carvalho J. (2021). Modeling, Assessment and Design of an Emergency Department of a Public Hospital through Discrete-Event Simulation. Appl. Sci..

[15] Hamza N., Majid M.A., Hujainah F. (2021). SIM-PFED: A Simulation-Based Decision Making Model of Patient Flow for Improving Patient Throughput Time in Emergency Department. IEEE Access.

[16] Tavakoli M., Tavakkoli-Moghaddam R., Mesbahi R., Ghanavati-Nejad M., Tajally A. (2022). Simulation of the COVID-19 Patient Flow and Investigation of the Future Patient Arrival Using a Time-Series Prediction Model: A Real-Case Study. Med. Biol. Eng. Comput..

[17] Al-Shareef A.S., Al Jabarti A., Babkair K.A., Jamajom M., Bakhsh A., Aga S.S. (2022). Strategies to Improve Patient Flow in the Emergency Department during the COVID-19 Pandemic: A Narrative Review of Our Experience. Emerg. Med. Int..

[18] Bovim T.R., Gullhav A.N., Andersson H., Dale J., Karlsen K. (2021). Simulating Emergency Patient Flow during the COVID-19 Pandemic. J. Simul..

[19] Louhab Z., Boufera F. (2022). Modelling and Simulation of Patient Flow in the Emergency Department During the COVID-19 Pandemic Using Hierarchical Coloured Petri Net. Int. J. Open Source Softw. Process. IJOSSP.

[20] Terning G., Brun E.C., El-Thalji I. (2022). Modeling Patient Flow in an Emergency Department under COVID-19 Pandemic Conditions: A Hybrid Modeling Approach. Healthcare.

[21] Helse Stavanger S. Nøkkeltall 2019/2020. https://helse-stavanger.no/om-oss/nokkeltall-20192020.

[22] Suh H. Om Oss. https://helse-stavanger.no/om-oss.

[23] Minge A. Hun Skal Hele Tiden Være Orakelet og ta Raske og Rette Avgjørelse. Denne Dagen Varte Pausen i 30 Sekunder. Stavanger Aftenblad 2020..

[24] Gallagher Healthcare What Are the Different Types of Hospitals?. https://www.gallaghermalpractice.com/blog/post/what-are-the-different-types-of-hospitals.

[25] Randers J. (1980). Elements of the Systems Dynamics Method.

[26] Terning G., Brun E. (2021). Systemic Conceptual Modeling of Patient Flow in a Hospital Emergency Department: A Case Example. Proceedings of the System Dynamics Society Record of the 38th International Conference of the System Dynamics Society.

[27] Brailsford S.C. Hybrid Simulation in Healthcare: New Concepts and New Tools. Proceedings of the 2015 Winter Simulation Conference (WSC).

[28] Lättilä L., Hilletofth P., Lin B. (2010). Hybrid Simulation Models—When, Why, How?. Expert Syst. Appl..

[29] Borshchev A. (2013). The Big Book of Simulation Modeling: Multimethod Modeling with Anylogic 6.

[30] Ronen B., Pliskin J.S., Pass S. (2018). The Hospital and Clinic Improvement Handbook: Using Lean and the Theory of Constraints for Better Healthcare Delivery.

[31] Asgary A., Najafabadi M.M., Karsseboom R., Wu J. (2020). A Drive-through Simulation Tool for Mass Vaccination during COVID-19 Pandemic. Healthcare.

[32] Gul M., Guneri A.F. (2015). A Comprehensive Review of Emergency Department Simulation Applications for Normal and Disaster Conditions. Comput. Ind. Eng..

[33] Ferrer J., Salmon M., Temime L. (2013). Nosolink: An Agent-Based Approach to Link Patient Flows and Staff Organization with the Circulation of Nosocomial Pathogens in an Intensive Care Unit. Procedia Comput. Sci..

